# CFTR Modulators: Impact on Fertility, Pregnancy, and Lactation in Women with Cystic Fibrosis

**DOI:** 10.3390/jcm9092706

**Published:** 2020-08-21

**Authors:** Jennifer L. Taylor-Cousar

**Affiliations:** National Jewish Health, Internal Medicine and Pediatrics, Pulmonary, Denver, CO 80206, USA; taylorcousarj@njhealth.org

**Keywords:** cystic fibrosis, cystic fibrosis transmembrane conductance regulator (CFTR), modulator, pregnancy, fertility, lactation

## Abstract

Cystic fibrosis (CF) is a life-shortening genetic disorder caused by mutations in the cystic fibrosis transmembrane conductance regulator (CFTR) gene. These mutations lead to abnormal ion transport in mucous membranes throughout the body, including in the respiratory and gastrointestinal and reproductive tracts. Improvements in care and therapy have led to substantial increases in the quantity and quality of life for those with CF. Consequently, women with CF are increasingly interested in having families. Although pregnancy was once discouraged for women with CF, at this point, even women with moderately severe lung disease can successfully navigate pregnancy. With the recent approval of a triple combination CFTR modulator therapy that improves lung function, nutritional status, and quality of life for people with a single copy of the most common CFTR mutation, it is expected that the number of women with CF who choose to become pregnant will continue to increase. Although animal reproduction models show no alarming signals for use during pregnancy at normal human doses, there is a paucity of human safety data in pregnancy and lactation. This review summarizes what is currently known about the impact of use of CFTR modulators on fertility, pregnancy, and lactation in women with CF.

## 1. Introduction

Cystic fibrosis, a genetic disorder observed in people of all races and ethnicities, affects approximately 80,000 people worldwide. It is caused by mutations in the cystic fibrosis transmembrane conductance regulator (CFTR) gene, which leads to abnormal ion transport in mucous membranes throughout the body. The resultant thick, dehydrated, pH imbalanced mucus adversely impacts the function of the respiratory, gastrointestinal, and reproductive tracts.

**Figure 1:**
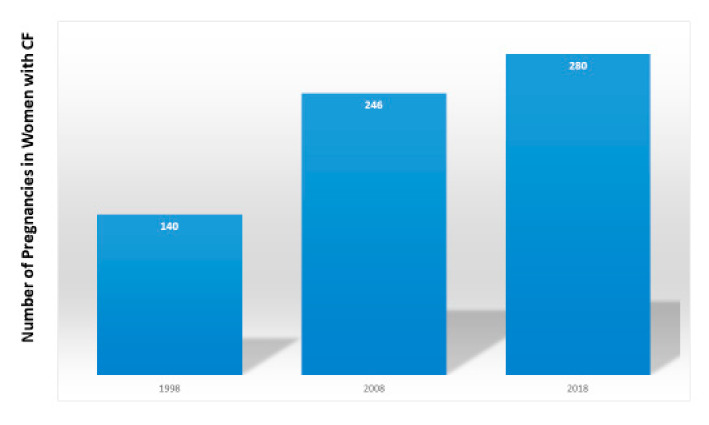
Number of pregnancies reported in the U.S. Cystic Fibrosis Foundation (CFF) Patient Registry [3] in women aged 14–years.

Since the first clinical and pathological descriptions of the disease in the 1930s [1,2], advances in care and the development of CF-specific therapies have led to an increase in survival. With the median predicted survival in the US now at 46 years, more than half of the people with CF are over the age of 18 years [3]. Not only has the quantity of people’s lives increased, but so has the quality. Thus, more women are expressing the desire to become pregnant [4]. The number of women with CF in the United States who became pregnant doubled from 140 in 1998 to 280 in 2018 [3] (Figure 1).

**Figure 2:**
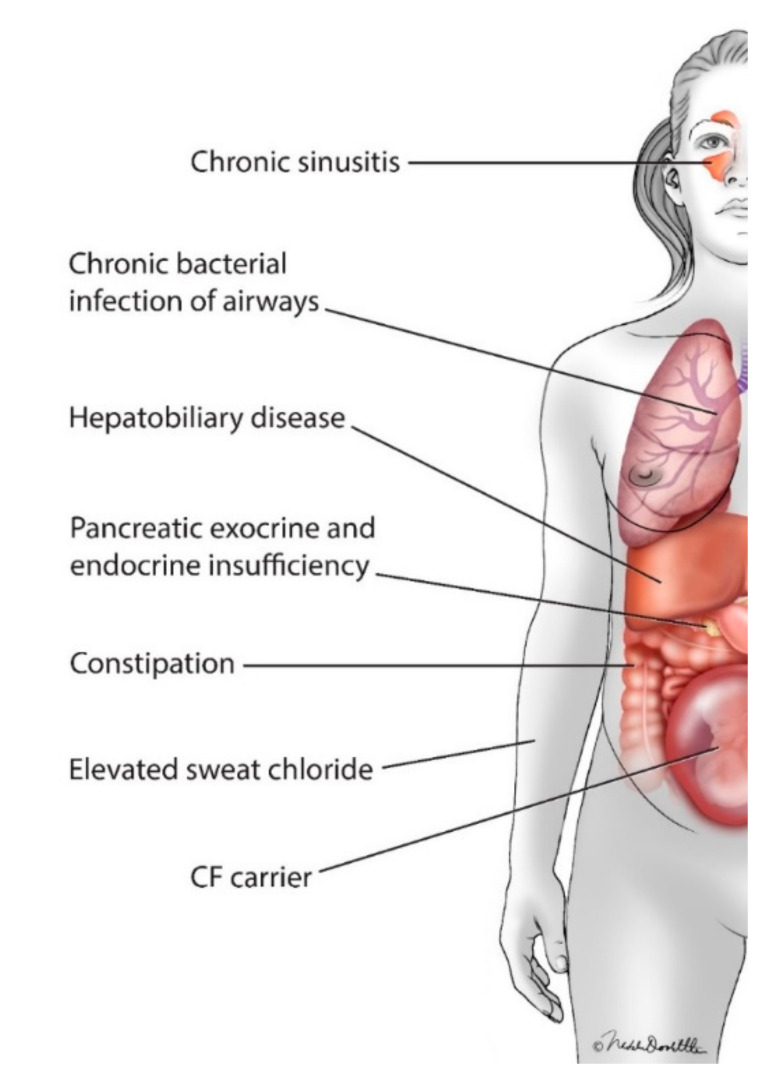
Women with cystic fibrosis (CF) who become pregnant and their infants may have increased risk because of the known complications that result from cystic fibrosis transmembrane conductance regulator (CFTR) dysfunction, including chronic respiratory infections, pancreatic insufficiency and CF-related diabetes.

The first pregnancy in a woman with CF was reported in the literature in 1960 [5]. Although the 20-year old carried the pregnancy to 34 weeks and delivered a healthy infant, she unfortunately died from her CF 6 weeks after the infant’s birth. Subsequently, multiple case series and single site retrospective studies suggested that, in spite of the multitude of consequences of CFTR dysfunction, some women with CF could successfully navigate pregnancy and motherhood, although lower lung function (as measured by percent-predicted forced expiratory volume in 1 s [ppFEV_1_]), infection with certain gram negative infections, such as the *Burkholderia cepacia* complex, and the complication of cystic fibrosis related diabetes (CFRD), were associated with increased risk [6,7,8,9,10,11] (Figure 2). More recent registry-based data suggests that pregnant women with CF and their infants experience more complications than pregnant women without CF [12,13]. Registry-based/large cohort studies demonstrated that, although pregnant women with CF may have increased illness-related visits and decreased quality of life, their survival was not decreased compared to that of women who did not become pregnant [14,15].

In addition to the potential risk of the common complications of CF to the health of women with CF and to that of their infants (including pancreatic endocrine and exocrine insufficiency and infectious sinus and pulmonary exacerbations; Figure 2), women with CF and their providers must also consider the potential risks of the many medications women with CF must take to maintain their health [16,17] versus the risk to the mother’s health of discontinuing the therapy [18,19]. Of particular importance for the care of women with CF is the use of the new class of pharmaceuticals, for which up to 90% of women with CF may be eligible: CFTR modulators.

CFTR modulators are the first pharmaceutical class designed specifically to alter the basic defect in CF by either improving the function of CFTR protein present at the cell surface and/or improving the trafficking of the CFTR to the cell surface [20,21]. The first approved CFTR modulator, ivacaftor, improved ppFEV_1_ by an average of 10.6% for the approximately 4% of people with the G551D (Gly551Asp) mutation [22,23]. Two more modestly effective modulator combinations were approved based on their ability to improve ppFEV_1_ and/or decrease CF pulmonary exacerbations [24,25,26]. In October 2019, the triple combination therapy, elexacaftor/tezacaftor/ivacaftor, which improved lung function by an average of 13.8% in people with one copy of F508del (Phe508del) and by 10% in people with CF already treated with tezacaftor/ivacaftor, was approved in the U.S. to treat people with CF with at least one copy of F508del [27,28].

This review summarizes what is currently known about the impact of use of CFTR modulators on fertility, pregnancy, and lactation in women with CF.

## 2. Impact of CFTR Modulators on the Sexual and Reproductive Health (SRH) of Women with CF

### 2.1. Impact of Modulators on Fertility

Tizzano and colleagues reported that there are variable levels of expression of CFTR in the cervical epithelium and fallopian tubes, but that post-puberty, the endometrial epithelium and glands express CFTR at high levels [29]. Women with CF generally have reproductive anatomy that is similar to that of women without CF, but abnormal CFTR function can lead to viscous, pH imbalanced cervical secretions that lead to subfertility in some women with CF [30]. Delayed puberty and ovulation, and suboptimal nutrition status may also play a role in decreased fertility. Recently, Shteinberg, et al. found older maternal age and exocrine pancreatic insufficiency to be risk factors for subfertility in women with CF [31].

Perhaps because of a personal history of infertility in the setting of poor health that later improved, or possibly because of misperceptions about female fertility based on the 97–98% incidence of male infertility in CF [32], 25–50% of pregnancies in women with CF are unplanned [33,34]. CFTR modulators in animal models showed no adverse impact on fertility when given at normal human doses [35,36,37,38] (Table 1). In people using CFTR modulators and consequent systemic improvement in their CFTR function (leading to improvements in nutritional status, and possibly improving cervical mucus consistency and pH) [30], there have been reports of both improved fertility (7/12 women who previously reported infertility became pregnant with an average time to conception, following ivacaftor initiation of 3.2 months [range 1–8.5]) and unplanned pregnancies [39,40,41]. In the phase 3 trial of ivacaftor, in which participants agreed to use contraception throughout the trial [23], approximately 2% of the women became pregnant [39]. It was not reported whether the pregnancies represented failure of a particular contraceptive method or failure to use contraception. To avoid unplanned pregnancies, CF teams should routinely counsel women who are starting CFTR modulators about the potential for increased fertility.

### 2.2. Impact of Modulators on Pregnancy

In pregnancy, the benefit of any medication to the mother must be weighed against the risk of administration to the fetus. While many medications used in people with CF have sufficient history of use so that they are deemed safe for use during pregnancy, CFTR modulators are not amongst them [16,17]. However, unlike other medications used in the chronic treatment of people with CF, acute destabilization [18,19] and one incident of death [18] have been reported with abrupt discontinuation of CFTR modulators [18,19].

Prior to 2015, the federal drug administration (FDA) categorized the risk of drugs during pregnancy as A-D or X based on the combination of data from animal reproduction studies and studies in pregnant women. However, in order to facilitate an informed discussion between providers and pregnant women, the FDA now requires that prescription drugs submitted for FDA approval after 30 June 2015, must follow the new pregnancy and lactation labeling rule (PLLR) [42]. Rather than reporting risk categories, information is based on the required sponsor information stating how much drug was administered to the animals used in reproduction models compared to the maximum recommended human dose (MRHD), and what happened to the fetus as a result.

In animals, placental transfer of individual modulators (none have been tested in combination) has been established [35,36,37,38]. While ivacaftor, tezacaftor, and elexacaftor, given at maternally toxic doses, caused minor abnormalities (decreased body weight in rats given ivacaftor at 7 times the MRHD, decreased body weight, delayed pinna attachment and eye opening in rabbits given tezacaftor at 1 times the MRHD, and decreased body weight in rats given ≥4 times the MRHD), none of the approved modulators impacted organogenesis at normal human doses [35,36,37,38] (Table 1). Although this animal data is reassuring, because of the lack of data in pregnant women, caution is advised for use in pregnancy, and some clinicians have advised women to discontinue use of modulators during pregnancy [40,41,43,44,45].

In addition to describing results of administration of drugs in animal reproduction models, sponsors are required to state whether there are adequate and well-controlled studies of the agent in pregnant women to determine if there is a drug-associated risk of major birth defects or miscarriage. As the impacts of drugs in pregnancy are evaluated, it is important to know baseline rates of major birth defects and miscarriages in the population being studied. Based on a large database study conducted in California between 2005–2008, the research showed that pregnancies of women with CF were more likely to be affected by congenital anomalies (14.3% vs. 6.4%, *p* = 0.005); in particular, a trend towards the occurrence of cardiac anomalies in infants of women with CF at a rate 7 times higher than in pregnancies of mothers without CF was observed (3.9% vs. 0.5%, *p* = 0.08). Because the first CFTR modulator was not approved until 2012 [35], this cohort preceded the modulator era and thus cannot be used to speculate about the impact of CFTR modulators on the occurrence of congenital anomalies. Although the U.S. Cystic Fibrosis Foundation Patient Registry (CFFPR) collects data regarding the use of CFTR modulators, it does not collect data on infants born to mothers of CF; thus, it is not possible to determine from the U.S. CFFPR [3] the rate of congenital malformations in infants born to women with CF in general nor the rate of congenital malformation in infants in association with CFTR modulator use.

There are no studies of the use of CFTR modulators in pregnant women with CF; however, several case reports have appeared in the literature that include women who unintentionally and intentionally became pregnant while being treated with ivacaftor and lumacaftor/ivacaftor [39,40,41,43,44,45,46] (Table 2). Of the six pregnancies reported in five women using ivacaftor, four of the infants were exposed to ivacaftor in all trimesters (the number of trimesters of exposure was not reported in one case, and in one case, a woman discontinued ivacaftor when it was determined that she was pregnant) [39,40,43,46]. No infant abnormalities were reported for any of the pregnancies. Infants were full term in three of the pregnancies (infant gestational age of twins born to one mother was not reported). For a mother with severe lung dysfunction, whose infants were exposed to ivacaftor during all three trimesters, both pregnancies resulted in the birth of premature infants (34–36 weeks of age) [43]. In the two reports of infants born to mothers who used lumacaftor/ivacaftor during all three trimesters, infants were born at 38 weeks (maternal ppFEV_1_ 90%) and 35 weeks (maternal ppFEV_1_ 43–46%) [44,45]. One infant had mild hyperbilirubinemia in the neonatal period, which resolved spontaneously [44]. In a third report of two infants born to the same mother, both infants were exposed to lumacaftor/ivacaftor throughout both pregnancies; no adverse impacts on the infants were reported. [41] Of the eleven infants exposed to modulators during pregnancy, only four had formal ophthalmologic exams; all were normal. Importantly, three attempts to discontinue CFTR modulators because of unknown safety in pregnancy resulted in pulmonary decline in three women, leading to resumption of therapy [43,44,45].

In addition to the case reports, which provided reassurance for normal infant outcomes following modulator exposure during pregnancy, Nash et al. recently reported the results of an international survey of CF center physicians regarding modulator use in women with CF during pregnancy [47]. Results from a total of 64 pregnancies in 61 women were summarized (31 pregnancies with ivacaftor exposure, 26 pregnancies with lumacaftor/ivacaftor exposure, and 7 pregnancies with tezacaftor/ivacaftor exposure). For maternal and infant complications, the physician providing care for the mother was asked to provide his/her opinion regarding whether the complication was related to modulator use. Use of CFTR modulators during part or all of the pregnancy resulted in two maternal complications that were deemed related to CFTR modulator therapy (one pulmonary exacerbation and one incident of acute myelocytic leukemia [AML] were attributed to lumacaftor-ivacaftor; there are no other reports in the literature of AML in association with CFTR modulator use). Critically, cessation of modulator therapy (based on the unknown risk to the fetus of modulators in pregnancy) resulted in clinical decline in nine women, prompting resumption of therapy during pregnancy. More than half of the infants in the study were exposed to modulators for all three trimesters. No modulator-related complications were reported in infants exposed in utero. The miscarriage rate for women with CF on modulator therapy was 4.7% [47], which was lower than the reported 10–15% miscarriage rate expected in the general population [48].

### 2.3. Impact of Modulators on Lactation

Although breast feeding by women with CF was historically discouraged because of concerns about sodium content and milk nutrient composition, subsequent work demonstrated normal electrolyte and protein content [49]. Currently, the major factors determining recommendations for lactation for women with CF are related to a woman’s ability to maintain her weight in spite of the high caloric demands of breast feeding and whether re-initiation of medications of unknown risk or potential harm to the infant are indicated for the mother’s health [50]. Although the CFTR modulator ivacaftor is now approved for infants with CF as young as 6 months of age [35], there is minimal human data demonstrating safety in lactation. Thus, caution is advised for use of all CFTR modulators during lactation [35,36,37,38].

Animal reproduction models have demonstrated the presence of individual modulators in breast milk [35,36,37,38] (Table 1). Importantly, although ivacaftor did not cause congenital anomalies when administered to rats at normal human doses, at doses resulting in the exposure of 0.25 times the MRHD, ivacaftor administration in 7 to 35-day old rat pups led to the development of non-congenital lens opacities (cataracts) [35]. Because cases of non-congenital lens opacities have been reported in pediatric patients treated with CFTR modulators, it is recommended that pediatric patients who are treated with these agents undergo baseline and follow-up ophthalmological examinations [35,36,37]. Thus, it is advisable that infants exposed to CFTR modulators *in utero* or through breast milk undergo formal examination for cataracts.

As noted above, there is limited human data on the use of CFTR modulators during lactation. In a woman taking lumacaftor/ivacaftor during pregnancy, Trimble et al. measured concentrations of both drugs in maternal and infant plasma, cord blood, and breast milk [44]. Both drugs were present in maternal plasma at expected concentrations, but were also present in cord blood (in cord blood, the presence of lumacaftor was higher than in maternal plasma and at equivalent levels to maternal plasma for ivacaftor), infant plasma, and breast milk (at low levels). Although there were fluctuations in the infant’s liver function test values, it was not clear whether the occasional mild elevations resulted from the low levels of lumacaftor and ivacaftor exposure from breast feeding or from a normal variation. The infant had a normal ophthalmologic exam. Similarly, all four infants who had formal ophthalmologic exams in the international survey reported by Nash et al. (*n* = total 27 infants exposed to modulators during lactation: iva, *n* = 13; lum/iva, *n* = 9; tez/iva *n* = 5) had normal exams [47].

## 3. Patient Perspective

I am a 28-year-old woman who is homozygous for F508del. I was diagnosed with CF at 10 months old due to failure to thrive and pneumonia. My first hospitalization occurred when I was 8 years old and, by high school, I was hospitalized every 6 months for pulmonary exacerbations. My health was declining, and I remember my doctor at the time guessing that I would have 10–15 years before I would need a lung transplant.

My whole life, I had hoped to become a mother, but I never counted on that being my reality. In August 2015, I was in the open-label portion of the Orkambi^®^ study. Much to the surprise of me and my husband, we found out we were pregnant. Because I was in the Orkambi^®^ study, I had to withdraw from the study once I became pregnant, and, based on a lack of data, my CF team chose not to start commercial Orkambi^®^. As a result of discontinuing Orkambi^®^, my lung function initially dropped but rebounded some. My health remained relatively stable and I only needed oral antibiotics a couple of times during the pregnancy. After being induced 10 days early and undergoing a thankfully non-eventful labor and delivery, our son was born at a healthy 6 lbs.

I didn’t feel strongly about breastfeeding when I was pregnant, but when my son was born, I wanted to try. Based on the advice of my CF team, I stayed off Orkambi^®^ to minimize risks. However, when my son was 9 months old, I experienced a pulmonary exacerbation, requiring intravenous antibiotics. After weeks of deliberating with my team, my son’s pediatricians (not all of whom agreed with me), and painstakingly scouring whatever little research existed, I decided to continue breastfeeding while on Orkambi^®^. We chose to check my son’s liver enzyme levels for a couple of months (which were normal). Eventually, I switched to Symdeko^®^ when it was approved, and my son weaned himself just before his third birthday.

When Trikafta^®^ was approved ahead of schedule in October 2019, I was thrilled but torn, as my husband and I had discussed the possibility of growing our family. I let my miracle drug wait in the cupboard for a couple of months while we continued to try for baby #2 because I didn’t want to start the drug to see its great gains only to have to stop when I got pregnant. However, in January 2020, after a viral illness and associated decrease in lung function, my CF team and I decided it was time to start Trikafta^®^. After starting, my lung function increased by 20%. At that point, I had almost no cough, a lot of energy, and my quality of life improved greatly.

After discussing my health and impact of Trikafta^®^ with my obstetrics and CF teams, we agreed that the benefits of Trikafta outweighed any potential unknown risk to the baby. My husband and I were given the go-ahead to continue trying for baby #2. Less than one month after starting Trikafta^®^, we found out we were pregnant. I am currently 28 weeks into this pregnancy and overall feeling great. I think, due to being on Trikafta^®^, I have much more energy and much less mucus. Navigating a high-risk pregnancy with a toddler in the midst of a pandemic has been a challenging experience to say the least. Although there will always be unknowns when journeying through CF and pregnancy and breastfeeding, I have peace knowing that we made the best decision with what information we have right now. I am grateful for the research and conversations that continue to happen in the CF community that will help people after me to make these huge and important decisions.

## 4. Conclusions

With the substantial gains in health experienced by people with CF over the last 20 years, the number of women who desire families and are becoming pregnant is increasing. It is likely that CFTR modulators increase fertility in women with CF, but the safety of their use in pregnancy and lactation is understudied. Animal reproduction models do not show alarming signals, and the sum of data from case reports and case series of use of modulators during pregnancy provides additional encouragement about the safety of modulators during pregnancy. Because of reports of acute deterioration in health following cessation of modulators, risks to the mother’s health due to discontinuation of modulators during pregnancy must be weighed carefully against the unknown risks to the fetus. If a mother chooses to continue using CFTR modulators during pregnancy and lactation, the development of non-congenital cataracts in juvenile rats and case reports in pediatric patients treated with modulators suggest the need for infant ophthalmologic exams. A prospective study of the use of CFTR modulators during pregnancy and lactation is greatly needed so that providers can offer informed discussions to women with CF who must make this difficult choice to continue or discontinue CFTR modulators prior to attempting pregnancy or during pregnancy. A Cystic Fibrosis Foundation Therapeutics funded study, maternal and fetal outcomes in the era of CFTR modulators (MAYFLOWERS) will prospectively evaluate the impact of the use of this class of drugs on the health of women with CF and their infants.

## Figures and Tables

**Table 1:** Data from animal reproduction models investigating the impact of CFTR modulator administration on sexual and reproductive health (SRH).

SRH Impact	Ivacaftor	Lumacaftor	Tezacaftor	Elexacaftor
**Fertility**	Impaired fertility and reproduction in male and female rats at toxic human doses (5-7X MRHD)	No effects on female or male fertility at toxic human doses	No effects on female or male fertility at toxic human doses	Impaired male and female fertility and reproduction at toxic human doses (6-7X MRHD)
**Chromosomal Abnormalities**	No genotoxicity	No genotoxicity	No genotoxicity	No genotoxicity
**Pregnancy/Teratogenicity**	Rats: At maternally toxic dose, did not impact survival or organ development; ↓fetal body weight	Rats and Rabbits: No significant effect at maternally toxic dose	Rats: No significant effect at maternally toxic dose	Rats: At maternally toxic dose, did not impact survival or organ development; ↓fetal body weight
	Rabbits: At maternally toxic dose, did not impact survival or organ development		Rabbits: At maternally toxic dose, ↓fetal body weight, early development delay in pinna detachment/eye opening	Rabbits: At maternally toxic dose, did not impact survival or organ development
**Lactation**	Present in breast milk	Present in breast milk	Present in breast milk	Present in breast milk
**Neonatal cataracts**	Cataracts observed at all doses in juvenile(7 to 35 days) rats	For combination therapy, see ivacaftor	For combination therapy, see ivacaftor	For combination therapy, see ivacaftor

Maximal recommended human dose (MRHD); data from the United States prescribing information (USPI) for ivacaftor [35], lumacaftor/ivacaftor [36], tezacaftor/ivacaftor [37], and elexacaftor/tezacaftor/ivacaftor [38].

**Table 2:** Case reports of modulators and pregnancy.

Case Report	Modulator	Mother’s Baseline ppFEV^1^	Mother’s Genotype	Trimesters Exposed	Infant Gestational Age (Weeks)	Infant Health	Lactation Use
Kaminski and Nazareth, 2015 [46]	iva	94	G551D/3272-26A > G	1–3	39	Normal	No
Jones and Walshaw, 2015 [39]	iva	75	F508del/G551D	1–3	38	Normal	Not reported
Jones and Walshaw, 2015 [39]	iva	“Normal”	F508del/G551D	Not reported	Twins, not reported	Not reported	Not reported
Ladores et al., 2017 [40]	iva	103	G551D/1585 2A > G	1	40	Normal	Not reported
Vekaria et al., 2019 [43]	iva	46	F508del/G551D	1–3 ^	36	Normal, Normal ophthalmologic exam	Not reported
		43#		1–3 #	34	Normal, Normal ophthalmologic exam	Not reported
Trimble et al., 2018 [44]	lum/iva	90	F508del/F508del	1–3 *	38	Mild hyperbilirubinemia (resolved), Normal ophthalmologic exam at 37 and 184 days	Yes
Mainz et al., 2019 [45]	lum/iva	52	F508del/F508del	1–3 ǂ	35	Normal, Normal ophthalmologic exam “during first year”	No
Ladores et al., 2020 [41]	lum/iva	“Improved by 6% on lum/iva”	F508del/F508del	1–3	Not reported	Normal	Not reported
				1–3 §	Not reported	Normal	Not reported

Ivacaftor (iva), lumacaftor (lum), percent predicted forced expiratory volume in 1 s (ppFEV_1_); ^ interrupted at five weeks for unknown safety in pregnancy but restarted at 10 weeks because of pulmonary decline following discontinuation; # the same woman had two separate pregnancies on iva; during the second pregnancy, a reduced dose of 150 mg daily was used, while 150 mg was used twice daily during trimesters 2 and 3; * interrupted at 13 weeks for unknown risk in pregnancy but restarted at 15 weeks because of pulmonary decline following discontinuation; ǂ interrupted at 10 weeks for unknown risk in pregnancy but restarted at 15 weeks because of pulmonary decline following discontinuation. § The same woman had two separate pregnancies on lum/iva.
